# Prevalence and determinants of malaria among children in Zambézia Province, Mozambique

**DOI:** 10.1186/s12936-017-1741-z

**Published:** 2017-03-09

**Authors:** James G. Carlucci, Meridith Blevins Peratikos, Charlotte B. Cherry, Melanie L. Lopez, Ann F. Green, Lazaro González-Calvo, Troy D. Moon, the Ogumaniha-SCIP Zambézia Consortium

**Affiliations:** 1 Vanderbilt Institute for Global Health, 2525 West End Avenue, Suite 750, Nashville, TN 37203 USA; 2 Division of Pediatric Infectious Diseases, Department of Pediatrics, Vanderbilt University Medical Center, Nashville, TN USA; 3 Department of Biostatistics, Vanderbilt University, Nashville, TN USA; 4 Friends in Global Health, Maputo, Mozambique; 5 World Vision US, Federal Way, WA USA

**Keywords:** Malaria, Mozambique, Fever, Infant, Child, Prevalence, Risk factors, Health services accessibility

## Abstract

**Background:**

Malaria is the leading cause of death among children in Mozambique. Prevalence and factors associated with malaria are not well studied among children in rural Zambézia Province. Whether prevalence of malaria varies across diverse districts within the province is unknown.

**Methods:**

A cross-sectional survey of female heads of household was conducted during April and May 2014, a period of peak malaria transmission. Data were collected on up to two randomly selected children aged 6–59 months per household. The outcome of interest was self-report of symptomatic malaria confirmed by diagnostic test in the past 30 days. Analyses accounted for the two-stage cluster sample design. Prevalence of symptomatic malaria was calculated for the province and three over-sampled focus districts—Alto Molócuè, Morrumbala, and Namacurra. Multivariable logistic regression of symptomatic malaria diagnosis included: district, age, sex, education, bed net use, urban setting, distance to health facility, income, roofing material, and pig farming.

**Results:**

Data were collected on 2540 children. Fifty percent were female, and the median age was 24 months. Sixty percent of children slept under bed nets the night prior to the survey, but utilization varied between districts (range 49–89%; p < 0.001). Forty-three percent of children reported fever in the past 30 days, 91% of those sought care at a health facility, 67% of those had either a malaria rapid diagnostic test or blood smear, and 67% of those had a positive test result and therefore met our case definition of self-reported symptomatic malaria. There were significant differences in prevalence of fever (p < 0.001), health-seeking (p < 0.001), and diagnostic testing (p = 0.003) between focus districts. Province-wide prevalence of symptomatic malaria was 13% and among focus districts ranged from 14% in Morrumbala to 17% in Namacurra (p < 0.001). Higher female caregiver education (OR 1.88; 95% CI 1.31–2.70), having fewer young children in the household (OR 1.25; 95% CI 1.01–1.56), and higher income (OR 1.56; 95% CI 1.11–2.22) were independently associated with having a child with symptomatic malaria.

**Conclusions:**

Self-reported symptomatic malaria is highly prevalent among children in Zambézia Province, Mozambique and varies significantly between diverse districts. Factors facilitating access to health services are associated with symptomatic malaria diagnosis. These findings should inform resource allocation in the fight against malaria in Mozambique.

## Background

Malaria is a mosquito-borne parasitic disease caused by several species of the *Plasmodium* parasite. Malaria typically manifests as a febrile illness that can be fatal if unrecognized, especially in young children. Approximately 3.2 billion people live in malaria endemic areas, and, in 2015, there were an estimated 214 million cases and 438,000 deaths attributable to the disease. The vast majority of these deaths occurred in sub-Saharan African countries, including Mozambique [1].

**Fig. 1:**
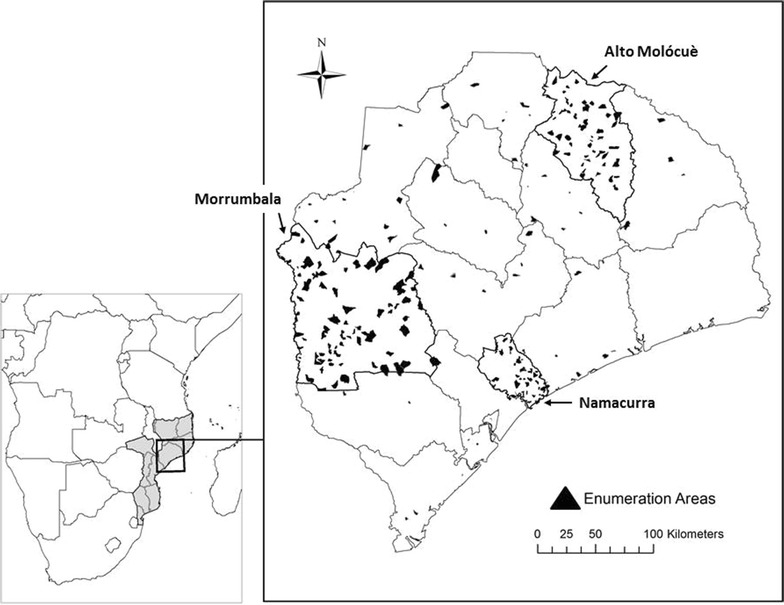
Map of Zambézia Province, Mozambique (*right panel*), as it is situated within Mozambique and Southern Africa (*left panel*). Borders of districts within the province are outlined, and the three oversampled focus districts (Alto Molócuè, Morrumbala, and Namacurra) are outlined in *bold*. Sampled enumeration areas are overlaid as *black polygons*. Charlotte Buehler Cherry; Vanderbilt Institute for Global Health; Projection: WGS 1984 Web Mercator Auxiliary Sphere. Permission has been granted by the copy-right holder for publication of this figure in an open access journal

Mozambique is a country in southeastern Africa where malaria is endemic. Zambézia Province is an impoverished rural province in the north-central part of Mozambique (Fig. 1). In Mozambique, malaria is the leading cause of death overall, accounting for approximately 30% of all deaths. The bulk of this mortality occurs in children less than 5 years of age [2].

Anopheles mosquitoes capable of transmitting malaria are found throughout Zambézia Province, but distribution may vary between districts based on environmental factors influencing mosquito suitability. Mosquitoes breed in standing water, and increased rainfall has been associated with increased incidence of malaria [3, 4]; thus, peak transmission of malaria should occur during the rainy season in Mozambique (December to April). Precipitation in Mozambique is highest in coastal districts like Namacurra (Fig. 1), which may result in an increased malaria risk [5]. Additionally, the relatively warmer temperatures in coastal districts may be more favorable for malaria transmission [4, 6]. Malaria is rarely found above 1500 m [4, 7], so the mountainous northern districts of Zambézia Province, such as Alto Molócuè (Fig. 1), may have pockets of protection from malaria [8].

Despite large-scale population-level investments to distribute malaria preventative measures across the districts of Zambézia Province, not all districts have benefited equally or according to perceived need. Campaigns to distribute insecticide treated bed nets (ITN) have been organized by the Mozambican Ministry of Health (MOH), the President’s Malaria Initiative (PMI), and the Global Fund/World Bank, but as of 2014 only 11 of 17 districts had received ITN initiatives. Alto Molócuè and Namacurra benefited from ITN distribution in 2012 and 2013, respectively. Meanwhile, Morrumbala (Fig. 1) had not yet benefited from ITN distribution [9]. Large disparities in ITN coverage across Zambézia Province have been reported, with 34% coverage in Morrumbala, compared to 90% coverage in Namacurra [10].

Indoor residual spraying (IRS), the practice of fumigating homes with insecticide to eliminate mosquitoes and prevent malaria, has also been differentially deployed across Zambézia Province since 2007 [9]. From the data available, it is difficult to precisely know which districts have benefited most from spraying campaigns, but the Mozambican MOH has reported that only 19% of households were sprayed in 2011 [11], while in 2013 PMI reported 89% IRS coverage in four districts of Zambézia Province, including Morrumbala but not Alto Molócuè or Namacurra [9].

Prevalence of malaria has not been well described in the rural provinces of Mozambique, nor has it been well studied among children. It has been estimated that prevalence of malaria in Zambézia Province might approach 55%, but this has not been disaggregated for children [11]. Whether malaria prevalence varies across diverse geographic regions/districts within the province is unknown. The aims of this research were: (1) to describe the prevalence of symptomatic malaria among children 6–59 months of age in Zambézia Province; (2) to determine whether prevalence of malaria among children varies between three distinct districts within the province—Alto Molócuè, Morrumbala, and Namacurra; and, (3) to identify factors associated with malaria among children in the province.

## Methods

### Study design

A cross-sectional, province-wide survey was conducted at the end of the rainy season in April and May 2014, representing a time of peak malaria transmission. This work was nested within a larger initiative, the *Ogumaniha* project, funded under the United States Agency for International Development (USAID) Strengthening Communities through Integrated Programming (SCIP) award (Cooperative Agreement 656-A-00-09-000141-00). The *Ogumaniha* project was broadly focused on improving the health and livelihood of children, women, and families in Zambézia Province [10, 12–15], and data collection for monitoring and evaluation mirrored these goals. Data elements relevant to malaria were used for this research.

### Sampling frame

The survey was province-wide, but not every person in the province was surveyed. At the time of the survey, there were approximately 3.8 million people living in 900,000 households in Zambézia Province. The province was further divided into 17 districts, each of which was further subdivided into enumeration areas (EA) for the purpose of census measurements. There were approximately 9000 EAs in Zambézia Province [11].

### Sampling methodology

To get from the sampling frame to individual level data, sampling was performed in several steps. First, the sampling frame was stratified by district, and a design weight was constructed to compensate for over-sampling in three focus districts selected for their diversity (Alto Molócuè, Morrumbala, and Namacurra). Then, EAs were selected for sampling within each stratum using probability proportional to size; EAs with a higher number of households had a proportionally greater probability of selection. Next, households within each EA were selected by dividing the EA into quadrants, identifying a central point within each quadrant, randomly selecting a direction in which to proceed, then choosing the first household in that direction as the starting point, and subsequently selecting the four nearest households. Finally, within each household, up to two children 6–59 months of age were selected based on randomly generated numbers corresponding to birth order.

### Population and sources of data

Once a household was selected, a mobile survey team would identify the female head of household. If she gave informed consent, then a face-to-face interview was conducted in her local language. A smart phone equipped with Open Data Kit software (University of Washington; Seattle, WA, USA) was used for data collection [16, 17]. The survey was conducted within the broader context of the *Ogumaniha* project, and as such it collected information on over 500 variables from a wide variety of topic areas including demographics, economic status, health knowledge, access to health services, and nutrition. The child health module was used for this research.

### Non-participation and non-response

Non-participation was defined as a person refusing to participate in the survey. Non-response was defined as refusal to answer particular survey questions. Efforts were made to minimize both non-participation and non-response, and ultimately 99.7% of planned surveys were completed. In the case of non-response these data were not missing at random, so to distinguish between missing data and refusal to respond, these were categorized as “don’t know” and “no response,” respectively.

### Sample summary

Ultimately, 262 EAs stratified by district grouping were sampled with probability proportional to size. The three focus districts were over-sampled to increase the precision of survey results and to facilitate comparisons between districts. 201 EAs were sampled from the three focus districts, and 61 EAs were sampled from the 14 remaining districts to maintain a degree of generalizability across the province (Fig. 1).

### Measurements

The main outcome of interest was self-reported symptomatic malaria, defined as female head of household report of a child with a febrile illness within the past 30 days, subsequently confirmed to be malaria by a diagnostic test. This outcome was assessed through a series of questions, which included, “Has your child had fever in the past 30 days? If so, did you seek care for your child at a health facility? If so, was a malaria diagnostic test performed [either smear or rapid diagnostic test (RDT)]? If so, did the test result confirm malaria?” If the respondent answered affirmatively to all of these questions, the child was classified as a case of symptomatic malaria.

The main exposure of interest was the district in which a child lived. This categorical variable was divided into the three focus districts (Alto Molócuè, Morrumbala, and Namacurra) and a fourth category for any other district within Zambézia Province.

Other exposures were treated as covariates and were considered as potential factors associated with symptomatic malaria. Child-specific covariates included: age, sex, whether the child slept under an ITN the night prior to the survey, and weight-for-age Z-score (WAZ). WAZ is calculated using age, weight, and sex, and scores are compared against World Health Organization (WHO) standards. Lower WAZ scores indicate a higher degree of undernutrition; a Z-score less than −1 is considered mild undernutrition, less than −2 is considered moderate undernutrition, and less than −3 is considered severe undernutrition [18]. Weight was collected in a random subset of EAs such that only 56% of children had WAZ.

Female head of household specific covariates included: age, level of education, understanding of Portuguese (the national language of Mozambique), and whether the woman slept under an ITN the night prior to the survey.

Household covariates included: number of persons in the household (household size), number of children less than 5 years of age in the household, monthly income [more or less than 1000 meticais (MZN) per month, which was roughly equivalent to one United States dollar per day in 2014], whether there was electricity in the home, number of ITNs in the home, distance to the nearest health facility (calculated from center of the EA to the health facility), rural versus urban setting, roofing material, and whether the family raised pigs. Thatch roofs are more permissive to mosquitoes and have been associated with malaria [19, 20]. Raising pigs has been associated with malaria, presumably due to nearby standing water [20].

### Mosquito suitability

A geographic information system (GIS)-based approach (ArcGIS 10.3.1; Esri; Redlands, California, USA) was used to create a map of mosquito suitability based on bioclimatic variables [21]. Review of mosquito habitat and suitability literature revealed temperature, precipitation, and elevation as important variables that modulate malaria suitability [3, 4, 6, 7, 22]. Administrative boundaries were obtained from DIVA-GIS Free Spatial Data (LizardTech, Inc.; Seattle, Washington, USA; and, the University of California, USA) [23]. Temperature and precipitation data were obtained from the Climate Research Unit v3.23 (University of East Anglia; Norwich Research Park, Norwich, United Kingdom) [24]; global land surface monthly mean time series datasets from 2011 to 2014 at 0.5° latitude/longitude grids were utilized [5]. Elevation data were obtained from GTOPO30 digital elevation model (U.S. Geological Survey’s EROS DataCenter; Sioux Falls, South Dakota, USA) at approximately 1 km resolution [8]. Temperature and precipitation data were resampled to 1 km resolution to match the elevation dataset resolution.

**Fig. 2:**
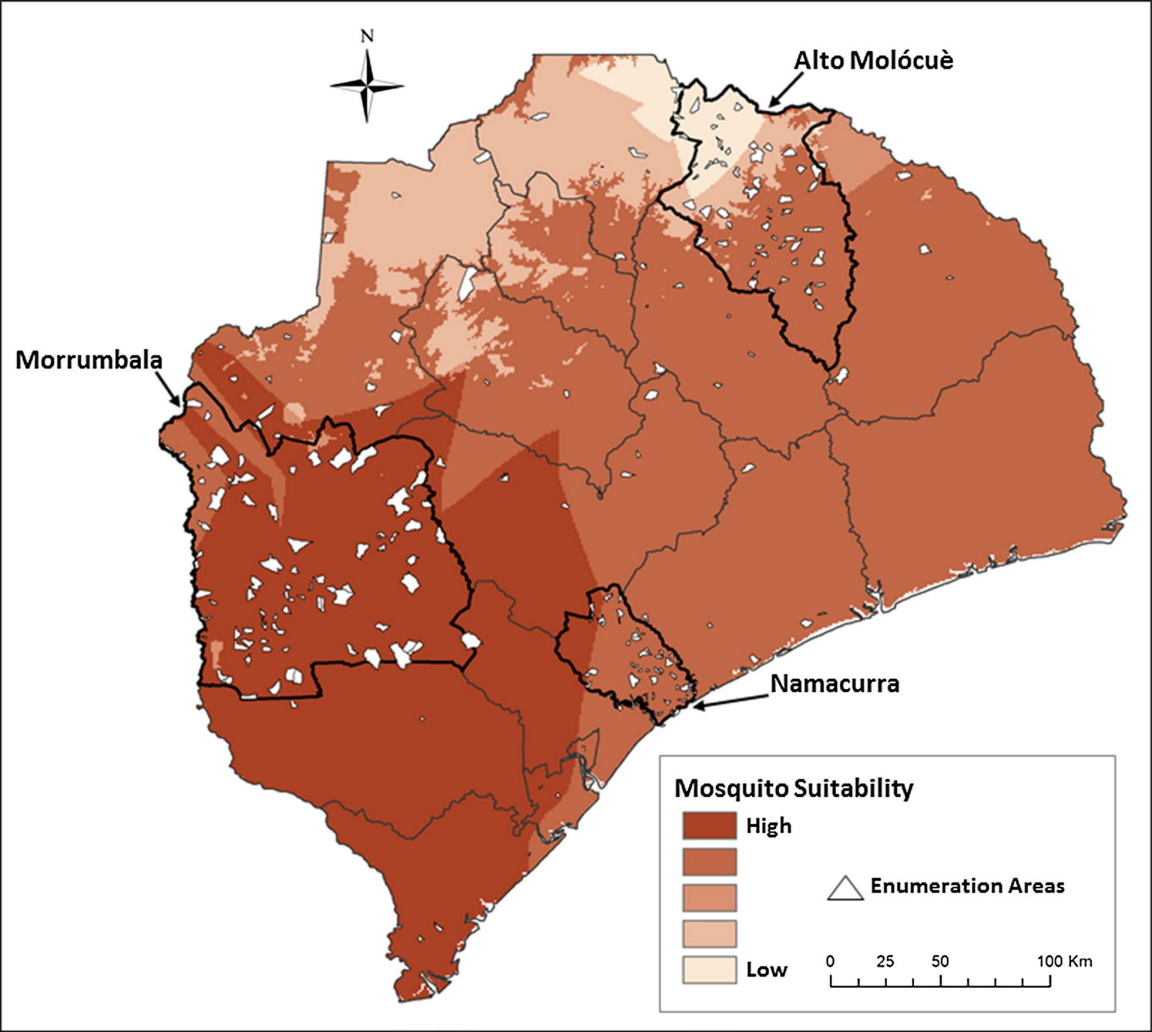
Predicted mosquito suitability in Zambézia Province, Mozambique. *Darker areas* are predicted to be more suitable for mosquitoes, and *lighter areas* are predicted to be less suitable for mosquitoes. Borders of districts within the province are outlined, and the three oversampled focus districts (Alto Molócuè, Morrumbala, and Namacurra) are outlined in *bold*. Sampled enumeration areas are overlaid as *white polygons*. Charlotte Buehler Cherry; Vanderbilt Institute for Global Health; Projection: WGS 1984 Web Mercator Auxiliary Sphere. Permission has been granted by the copy-right holder for publication of this figure in an open access journal

A weighted overlay method was applied to each of the three source data layers. Reclassification of each source layer was based on predicted mosquito suitability. Level 1 represented highest suitability and level 5 represented lowest suitability. Because elevation is an important variable modulating malaria risk, elevation was given an influence weight of 50% in the model, with temperature and precipitation given an influence weight of 25% each (Table 1). Weighted suitability values were overlaid and summed producing an overall mosquito suitability map (Fig. 2).

**Table 1:** Approach to weighted overlay method for creating a predicted mosquito suitability model for Zambézia Province, Mozambique

Mosquito suitability score	Elevation (m)	Mean annual temperature (°C)	Mean annual precipitation (mm)
1 = high suitability 5 = low suitability	Weight in model: 50%	Weight in model: 25%	Weight in model: 25%
1	≥650	>27	>271.9
2	Not applicable (NA)	26–27	257.5–271.9
3	NA	25–26	230.4–257.5
4	NA	24–25	201.1–230.4
5	<650	<24	<201.1

### Statistical analyses

Descriptive statistics were used to summarize attributes of respondent female heads of household, their children, and their households. These were reported for the entire province and stratified by the over-sampled focus districts. Continuous variables were reported as weighted estimates of the median and categorical variables as weighted percentages, with each observation weighted by the inverse of the household or child sampling probability.

Categorical responses from respondents regarding their child having fever and related behaviors/outcomes were reported as weighted percentages. Prevalence of symptomatic malaria was calculated as the weighted proportion of children with fever and a positive malaria diagnostic test. Comparisons between focus districts were made using Chi square tests, ignoring the effects of clustering. Data were reported for the entire province and stratified by over-sampled districts.

**Fig. 3:**
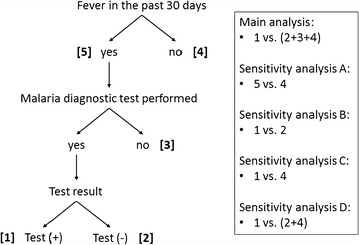
Schematic of multivariable logistic regression analyses to identify factors associated with self-reported symptomatic malaria among children in Zambézia Province, Mozambique. The main analysis compared those who had symptomatic malaria confirmed by a diagnostic test to the rest of the children in the cohort. Sensitivity analyses were conducted to account for the potential influences of accessibility of care and health-seeking behaviour

Multivariable logistic regression with robust variance estimation to account for clustering within households and EA was used to identify factors associated with self-reported symptomatic malaria among children. The main model compared those who had symptomatic malaria confirmed by a diagnostic test to the rest of the children in the cohort (Fig. 3). Covariates were selected based on a priori hypotheses and after checking for co-linearity and missingness. Where there was evidence of non-linearity of continuous covariates, variables were modeled using restricted cubic splines. To avoid overfitting, there were at least 15 malaria cases for every degree of freedom. Multiple imputation was used to account for missing survey responses in covariates. Outcomes were not imputed. Odds ratios (OR) with 95% confidence intervals (CI) were reported.

Anticipating that accessibility of care and health-seeking behavior might influence the main outcome of symptomatic malaria, and therefore the results of the main regression analysis, several additional sensitivity regression analyses were also performed. Children with fever in the past 30 days (regardless of whether they sought care or received a diagnostic test) were compared to those without report of fever (sensitivity analysis A; Fig. 3). Children who met the definition of symptomatic malaria were compared to other symptomatic children who accessed care and were tested for malaria but had a negative test result (sensitivity analysis B; Fig. 3). Children who had symptomatic malaria were compared to those who did not have fever and therefore did not access care or have a malaria diagnostic test (sensitivity analysis C; Fig. 3). Finally, children with symptomatic malaria were compared to a group that included both those who were symptomatic but had a negative malaria diagnostic test and those who did not have fever and therefore did not seek care or malaria testing (i.e., the comparison group excluded those who were symptomatic but not tested for malaria) (sensitivity analysis D; Fig. 3). Like the main model, all sensitivity analyses accounted for the sample design.

To determine whether observed prevalence of self-reported symptomatic malaria among children correlated with predicted mosquito suitability in Zambézia Province, each EA was assigned a mosquito suitability score (values of 1–5 as described in the “Mosquito suitability” section, above) corresponding to the suitability zones (Fig. 2). The relationship between EA-level mosquito suitability score and EA-level prevalence of symptomatic malaria was estimated using robust ordinary least squares regression. Similarly, robust ordinary least squares regression was used to determine whether EA-level mosquito suitability score was correlated with EA-level prevalence of reported fever among children.

R version 3.2.5 (Free Software Foundation, Inc.; Boston, Massachusetts, USA) [25] and ArcGIS 10.3.1 (Esri; Redlands, California, USA) [21] were used for data analyses.

## Results

Data were collected on 2540 children aged 6–59 months in April and May 2014. Sixty-eight percent of these children were from the three focus districts—Alto Molócuè, Morrumbala, and Namacurra. Fifty percent were female, and the median age was 24 months. Weights were not documented for 44%, but among those with anthropometric measurements, 35% were undernourished. Among respondent female heads of household, median age was 26 years, 77% had only 0–5 years of education, and 47% understood Portuguese well. Median household size was five persons, the majority of households had 1–2 children less than 5 years of age, and 83% of households earned less than 1000 MZN per month. Seventy-eight percent of homes were in rural areas, 79% of homes had thatched roofs, and only 11% of homes had electricity. Demographics and characteristics were similar across focus districts. Notable exceptions were that respondent level of education and understanding Portuguese were especially low in Morrumbala, and monthly household income was higher in Alto Molócuè than in Morrumbala and Namacurra (Table 2).

**Table 2:** Characteristics of children, female head of household respondents, and households in Zambézia Province and across three focus districts

	All Province	Alto Molócuè	Morrumbala	Namacurra
	(n = 2540)	(n = 781)	(n = 522)	(n = 416)
Sex of child				
Male (%)	50.2	47.5	43.3	49.2
Female (%)	49.8	52.5	56.7	50.8
Age of child (months)	24 (12–36)	24 (12–36)	24 (12–36)	24 (12–36)
Weight (kg)	10.9 (9.2–13)	10.2 (8.5–12.1)	10.2 (8.5–12.5)	11 (9.5–13.4)
Missing, n (%)	1119 (44)	596 (76)	313 (60)	153 (37)
Weight for age WHO Z-score				
Missing, n (%)	1191 (47)	604 (77)	331 (63)	169 (41)
≤−3 (%)	4.6	1.4	5.6	5.3
−2.99 to −2 (%)	10.5	9.2	10.3	8.9
−1.99 to −1 (%)	19.9	29.9	16.9	19.1
>−1 (%)	65.0	59.5	67.2	66.7
Age of respondent (years)	26 (22–33)	26 (22–33)	28 (22–38)	27 (21–33)
Missing, n (%)	120 (5)	13 (2)	51 (10)	26 (6)
Education of respondent (years)	3 (0–5)	4 (2–6)	1 (0–2)	2 (0–5)
Education of respondent (category) (years)				
0–5 (%)	77.2	73.0	93.1	77.3
6–10 (%)	18.7	25.9	6.6	20.3
>10 (%)	4.1	1.1	0.3	2.3
Respondent understands Portuguese (%)	47.3	63.5	19.0	40.1
Urban/rural				
Rural (%)	77.5	89.7	96.0	100.0
Urban (%)	22.5	10.3	4.0	0.0
Household size	5 (4–6)	5 (4–6)	5 (4–6)	4 (4–5)
Number of children under 5 years	2 (1–2)	2 (1–2)	2 (1–2)	2 (1–2)
Ethnic group^a^				
Elomwe (%)	27.8	52.5	2.0	0.5
Echuabo (%)	12.1	0.1	1.3	81.2
Cisena (%)	7.1	0.0	67.2	0.2
Cinyanja (%)	10.1	0.0	0.9	0.0
Other (%)	7.2	0.0	11.4	0.5
Missing	895 (35%)	375 (48%)	126 (24%)	85 (20%)
Household income (sum of all members’ monthly earnings)				
Missing, n (%)	613 (24)	58 (7)	90 (17)	133 (32)
<1000 MZN per month (%)	82.6	63.4	75.5	92.9
≥1000 MZN per month (%)	17.4	36.6	24.5	7.1
Roofing material				
Missing, n (%)	784 (31)	283 (36)	187 (36)	139 (33)
Thatch (%)	78.7	84.8	93.3	83.2
Other (%)	21.3	15.2	6.7	16.8
Electricity in the home (%)	11.4	7.5	4.0	0.7
Does the family raise pigs (%)	9.2	28.4	32.7	1.1
Missing, n (%)	128 (5)	21 (3)	9 (2)	18 (4)
EA distance to health facility (km)	9.3 (4–16.8)	11.8 (6.1–17.8)	10.7 (5.5–20.5)	6.2 (4–8)

Continuous variables are reported as weighted estimates of median (interquartile range), with each observation being weighted by the inverse of the child sampling probability

Categorical variables are reported as weighted percentages, with each observation being weighted by the inverse of the child sampling probability

^a^Respondents were allowed to select all that apply, so totals may add up to greater than 100%. Missing indicates no category was selected or documented

Sixty percent of children and 55% of respondents slept under an ITN the night prior to the survey. However, ITN utilization varied between focus districts (p < 0.001), with the highest utilization in Namacurra where 88% of children and 87% of respondents slept under an ITN the night prior to the survey, and with the lowest utilization in Morrumbala where 49% of children and 38% of respondents slept under an ITN the night prior to the survey. Fifty-three percent of households in Morrumbala reported that there was not a single ITN in the home, while 20 and 7% of homes did not have at least one ITN in Alto Molócuè and Namacurra, respectively (p < 0.001) (Table 3).

**Table 3:** Bed net availability and utilization in Zambézia Province and across three focus districts

	All Province	Alto Molócuè	Morrumbala	Namacurra	p value*
	(n = 2540)	(n = 781)	(n = 522)	(n = 416)	
How many mosquito nets in the household					<0.001
Missing, n (%)	77 (3)	13 (2)	16 (3)	9 (2)	
Less than the number of beds (%)	38.2	40.8	39.0	37.1	
More than the number of beds (%)	3.6	5.2	0.3	8.3	
None (%)	33.8	20.1	53.1	7.3	
One for every bed (%)	24.4	33.9	7.6	47.3	
Child slept under bed night last night					<0.001
Missing, n (%)	156 (6)	81 (10)	20 (4)	5 (1)	
No (%)	40.4	34.3	51.5	11.6	
Yes (%)	59.6	65.7	48.5	88.4	
Respondent slept under bed net last night					<0.001
Missing, n (%)	59 (2)	20 (3)	8 (2)	10 (2)	
No (%)	44.7	39.9	61.7	12.8	
Yes (%)	55.3	60.1	38.3	87.2	

Categorical variables are reported as weighted percentages, with each observation being weighted by the inverse of the child sampling probability

* Chi square tests comparing across three focus districts. p values ignore effects of clustering

Among children with report of whether or not they had fever in the 30 days prior to the survey (n = 2501), 43% had a febrile illness. Fever was significantly more common among children from Namacurra (49%) and Alto Molócuè (46%) than in Morrumbala (38%) (p < 0.001). Among those who reported fever in the past 30 days, 73% sought advice and/or treatment for the fever. In Namacurra 93% of those seeking care went to a health facility, compared to 87 and 86% in Alto Molócuè and Morrumbala, respectively (p < 0.001). Eight percent of those seeking care in Morrumbala visited a traditional healer, while 3 and 4% did so in Alto Molócuè and Namacurra, respectively (p < 0.001). Among those seeking care at a health facility, 67% had at least one malaria diagnostic test performed. Both RDTs and blood smears were more commonly performed in Alto Molócuè than in other focus districts (p = 0.003). Among those who had any malaria diagnostic test performed, 67% had a positive test result, consistent with a diagnosis of self-reported symptomatic malaria. Overall, the prevalence of symptomatic malaria among children in Zambézia Province was 13%, and prevalence varied significantly between focus districts, with the highest prevalence in Namacurra (17%) and the lowest prevalence in Morrumbala (14%) (p < 0.001) (Table 4).

**Table 4:** Prevalence of fever, healthcare utilization, and malaria among children in Zambézia Province and across three focus districts

	All Province	Alto Molócuè	Morrumbala	Namacurra	p value*
	(n = 2540)	(n = 781)	(n = 522)	(n = 416)	
Fever in the past 30 days					<0.001
Missing, n (%)	39 (2)	24 (3)	4 (1)	1 (<1)	
No (%)	56.6	53.6	61.6	50.6	
Yes (%)	43.4	46.4	38.4	49.4	
Sought advice or treatment for the fever (if fever)	72.5%	78.4%	79.2%	69.6%	0.47
Source of medical care (if sought advice)					<0.001
Missing, n (%)	269 (25)	75 (21)	49 (23)	56 (28)	
Family member (%)	2.8	7.6	3.0	0.0	
Health facility (%)	91.3	87.1	86.0	93.1	
Other (%)	1.1	2.0	3.1	1.1	
Pharmacy (%)	0.1	0.8	0.0	2.1	
Traditional healer (%)	4.7	2.5	8.0	3.8	
Malaria diagnostic performed (if health facility)					
Rapid diagnostic test (RDT)					0.003
Missing, n (%)	19 (3)	14 (6)	0 (0)	0 (0)	
No (%)	34.5	25.8	32.5	35.0	
Yes (%)	65.5	74.2	67.5	65.0	
Blood smear					0.025
Missing, n (%)	27 (4)	17 (7)	1 (1)	2 (2)	
No (%)	41.4	32.0	35.0	41.1	
Yes (%)	58.6	68.0	65.0	58.9	
Either RDT or smear					0.003
Missing, n (%)	19 (3)	14 (6)	0 (0)	0 (0)	
No (%)	33.4	24.2	28.6	34.4	
Yes (%)	66.6	75.8	71.4	65.6	
Positive test result for malaria (if test performed)					0.18
Missing, n (%)	4 (1)	1 (1)	0 (0)	2 (2)	
No (%)	32.7	33.6	30.4	19.6	
Yes (%)	67.3	66.4	69.6	80.4	
Estimated prevalence of malaria	12.8%	15.9%	13.6%	16.8%	<0.001

Continuous variables are reported as weighted estimates of median (interquartile range), with each observation being weighted by the inverse of the child sampling probability

Categorical variables are reported as weighted percentages, with each observation being weighted by the inverse of the child sampling probability

* Chi square tests comparing across three focus districts. p values ignore effects of clustering

In the multivariable regression model, which included all children with a response to whether or not they had fever in the 30 days prior to the survey (n = 2501), having more years of female caregiver education (OR 1.88; 95% CI 1.31–2.70), fewer children less than 5 years of age in the household (OR 1.25; 95% CI 1.01–1.56), and household income of 1000 MZN or more per month (OR 1.56; 95% CI 1.11–2.22) were independently associated with self-reported symptomatic malaria. In this adjusted model, odds of symptomatic malaria were similar between focus districts. In the sensitivity analysis restricted to children who accessed care at a health facility and who were tested for malaria (sensitivity analysis B; n = 533), caregiver education (OR 1.02; 95% CI 0.63–1.64), number of young children in the household (OR 1.38; 95% CI 0.94–2.04), household income (OR 1.45; 95% CI 0.74–2.78), and district in which the child lived [(Alto Molócuè, OR 1.00 (reference)); (Morrumbala, OR 1.38; 95% CI 0.69–2.77); (Namacurra, OR 2.12; 95% CI 0.95–4.74); (other district, OR 1.14; 95% CI 0.61–2.13)], were not associated with malaria diagnosis. The other sensitivity analyses (A, C, and D) yielded results similar to the main regression model (Table 5).

**Table 5:** Multivariable logistic regression analyses to identify factors associated with self-reported symptomatic malaria among children in Zambézia Province, Mozambique with adjusted odds ratios (OR) and 95% confidence intervals (CI) reported

	Main analysis^a^ OR (95% CI)	Sensitivity analysis A^b^ OR (95% CI)	Sensitivity analysis B^c^ OR (95% CI)	Sensitivity analysis C^d^ OR (95% CI)	Sensitivity analysis D^e^ OR (95% CI)
District					
Alto Molócuè (reference)	1	1	1	1	1
Morrumbala	1.24 (0.76, 2.02)	0.88 (0.61, 1.26)	1.38 (0.69, 2.77)	1.16 (0.69, 1.96)	1.21 (0.73, 1.99)
Namacurra	1.40 (0.88, 2.24)	1.38 (0.98, 1.96)	2.12 (0.95, 4.74)	1.50 (0.91, 2.47)	1.54 (0.95, 2.51)
Others	0.86 (0.55, 1.34)	0.93 (0.69, 1.24)	1.14 (0.61, 2.13)	0.87 (0.55, 1.38)	0.89 (0.57, 1.40)
Age (per 6 months)	1.04 (0.95, 1.14)	0.94 (0.88, 1.00)	1.82 (0.99, 3.36)	1.00 (0.92, 1.09)	1.01 (0.93, 1.11)
Male vs. female child	0.96 (0.75, 1.24)	1.00 (0.86, 1.18)	0.95 (0.62, 1.46)	0.99 (0.76, 1.28)	0.98 (0.76, 1.28)
Weight (per 1 kg)	0.87 (0.60, 1.26)	0.94 (0.73, 1.21)	0.79 (0.49, 1.28)	0.88 (0.61, 1.28)	0.87 (0.60, 1.25)
Respondent age (per 10 years)	1.05 (0.87, 1.27)	1.01 (0.88, 1.16)	1.15 (0.85, 1.54)	1.05 (0.87, 1.27)	1.07 (0.89, 1.28)
Respondent education (5 vs. 0 years)	1.88 (1.31, 2.70)	1.30 (0.96, 1.74)	1.02 (0.63, 1.64)	1.94 (1.31, 2.85)	1.96 (1.35, 2.85)
Respondent understands Portuguese	1.15 (0.83, 1.61)	1.06 (0.81, 1.38)	1.02 (0.58, 1.79)	1.15 (0.78, 1.68)	1.10 (0.77, 1.59)
Rural vs. urban	0.60 (0.36, 1.02)	0.81 (0.48, 1.35)	0.77 (0.34, 1.73)	0.60 (0.34, 1.06)	0.61 (0.35, 1.06)
Household size (per 1 member)	1.03 (0.94, 1.12)	1.07 (1.01, 1.15)	1.06 (0.91, 1.24)	1.04 (0.96, 1.14)	1.05 (0.97, 1.15)
Children under 5 (per 1 less child)	1.25 (1.01, 1.56)	1.01 (0.87, 1.18)	1.38 (0.94, 2.04)	1.22 (0.98, 1.54)	1.25 (0.99, 1.59)
Monthly income ≥1000 MZN	1.56 (1.11, 2.22)	1.18 (0.87, 1.59)	1.45 (0.74, 2.78)	1.59 (1.11, 2.27)	1.59 (1.12, 2.22)
Roofing material					
Thatch (reference)	1	1	1	1	1
Other	1.00 (0.56, 1.79)	1.25 (0.79, 1.99)	0.75 (0.34, 1.67)	1.11 (0.56, 2.20)	1.09 (0.57, 2.09)
House has electricity	0.68 (0.37, 1.23)	0.60 (0.37, 0.97)	0.58 (0.23, 1.47)	0.57 (0.32, 1.04)	0.58 (0.32, 1.07)
Household raises pigs	1.16 (0.80, 1.68)	1.10 (0.81, 1.50)	1.10 (0.60, 2.01)	1.17 (0.77, 1.79)	1.17 (0.78, 1.77)
Distance of enumeration area to health facility (20 vs. 5 km)	1.03 (0.87, 1.21)	1.04 (0.89, 1.22)	1.27 (0.95, 1.71)	1.03 (0.85, 1.25)	1.05 (0.87, 1.25)
Child slept under bed net the night prior to survey	1.28 (0.86, 1.92)	1.15 (0.85, 1.56)	1.24 (0.68, 2.23)	1.30 (0.86, 1.97)	1.31 (0.87, 1.96)
Respondent slept under bed net the night prior to survey	0.98 (0.64, 1.51)	0.87 (0.66, 1.15)	1.32 (0.70, 2.48)	0.95 (0.62, 1.47)	0.97 (0.64, 1.49)

Missing values of covariates were accounted for using multiple imputation in the main and sensitivity analyses

Because there was evidence (p < 0.10) that the relationship with log-odds of symptomatic malaria is non-linear, respondent education was fit using restricted cubic splines in the in the main and sensitivity analyses

^a^Main analysis: compares those who had symptomatic malaria confirmed by a diagnostic test to the rest of the children in the cohort. There are 2501 children included in this model, 380 of which had symptomatic malaria. There are 18 symptomatic malaria cases for every degree of freedom. The main model was repeated excluding child weight, due to excessive missingness, and results were similar. Respondent education is associated with household income (median [interquartile range] education among those with household income <1000 MZN per month was 3 [0–5] years, and among those with household income ≥1000 MZN per month was 4 [1–6] years). When household income was removed from the model, the OR (95% CI) for 5 vs. 0 years of education was 1.93 (1.32, 2.83). When education was removed from the model, the OR (95% CI) for household income ≥1000 MZN per month was 1.61 (1.15, 2.27)

^b^Sensitivity analysis A: compares those who reported fever in the past 30 days (cases) to those without report of fever. There are 2501 children included in this model, 1092 of which reported fever in the past 30 days

^c^Sensitivity analysis B: compares those who had symptomatic malaria confirmed by a diagnostic test to other symptomatic children who accessed care and were tested for malaria but had a negative test result. There are 533 children included in this model, 380 of which had symptomatic malaria

^d^Sensitivity analysis C: compares those who had symptomatic malaria confirmed by a diagnostic test to those who did not have fever and therefore did not access care or have a malaria diagnostic test. There are 1789 children included in this model, 380 of which had symptomatic malaria

^e^Sensitivity analysis D: compares those who had symptomatic malaria confirmed by a diagnostic test to a group that included both those who were symptomatic but had a negative malaria diagnostic test and those who did not have fever and therefore did not seek care or malaria testing (i.e., the comparison group excluded those who were symptomatic but not tested for malaria). There are 1942 children included in this model, 380 of which had symptomatic malaria

No significant associations were observed between geospatial prevalence of symptomatic malaria and predicted mosquito suitability (beta estimate −0.016 [95% CI −0.038, 0.007]; p = 0.18) or between prevalence of fever and predicted mosquito suitability (beta estimate 0.003 [95% CI −0.024, 0.030]; p = 0.82).

## Discussion

In this large cross-sectional survey, self-reported symptomatic malaria was highly prevalent among children 6–59 months of age in Zambézia Province, Mozambique. In unadjusted comparisons, prevalence of symptomatic malaria significantly varied between the diverse oversampled focus districts, with highest prevalence in the coastal district of Namacurra and lowest prevalence in the inland western district of Morrumbala. However, in adjusted comparisons, there was little evidence of a link between district of residence and malaria. Instead, higher levels of female head of household education, higher household income, and having fewer young children in the household were independently associated with self-reported symptomatic malaria. These findings suggest that self-reported symptomatic malaria may be influenced by factors facilitating access to health care services, and they highlight the need for intensified focus on the poorest and least educated caregivers, who may have the most difficulty seeking malaria diagnosis, care, and treatment for their children.

Interestingly, of the three focus districts, Morrumbala had the lowest observed symptomatic malaria, despite having the highest mosquito suitability (Fig. 2) and the lowest availability and utilization of ITNs (Table 3). Low levels of ITN ownership and use in Morrumbala have been reported in prior research on ITN use in Zambézia Province [10], and the fact that mass ITN distribution campaigns had not yet reached Morrumbala at the time of this research, likely contributed [9]. Considering that ITNs are highly effective at preventing mosquitoes from transmitting malaria and at reducing childhood morbidity and mortality from malaria [26], it is surprising that higher prevalence of symptomatic malaria was not reported in Morrumbala. That said, this study did not specifically measure or question about other known mosquito vector prevention measures being implemented in the province, such as IRS. As previously described, we know that IRS campaigns were being performed around the time of our survey implementation and that Morrumbala disproportionately benefitted relative to Alto Molócuè and Namacurra [9].

Furthermore, prior research has demonstrated that ITN use by children and female head of household respondents was associated with higher levels of respondent education and higher household income [10], the same factors that were associated with self-report of symptomatic malaria in this study. Therefore, it is possible that these factors broadly facilitate access to health services, including both ITNs and malaria diagnostics. This would be consistent with other studies that highlight the importance of empowering women with the education and resources that they need to ensure optimal health care for their children [27–32] and would support the United Nations Sustainable Development Goals, which emphasize poverty solutions and quality education for women [33]. That the effects of education and income were no longer evident in a sensitivity analysis restricted only to those who accessed care and had diagnostic testing performed further supports the possibility that this assessment of self-reported symptomatic malaria may be influenced by factors facilitating access to health care services.

Relying on respondent report of fever and malaria diagnostic test results over the past 30 days might also be prone to recall and/or reporting bias, and the extent to which bias may have occurred could have been affected by maternal education and may have differed between education strata. An alternative approach to assessing malaria prevalence would be to perform malaria testing at the same time as the survey, as has been done in other assessments of malaria prevalence in Mozambique [19, 20, 34–38]. That said, coordinating this survey with malaria testing would have been much more resource intensive, and as such was not possible for this research, which was nested within a larger initiative broadly focused on community development. Furthermore, not every case of parasitaemia is symptomatic; some people, especially those who have previously been infected by malaria and those with HIV infection, may asymptomatically harbour the malaria parasite [38]. This research instead focused on identifying symptomatic malaria, the hallmark of which is fever.

Malaria is the leading cause of fever in sub-Saharan Africa, where 30–60% of fevers during the rainy season are attributable to malaria [34, 39]. The proportion of fever attributable to malaria is even higher in infants and younger children [34]. In highly endemic areas, fever alone has been used as a surrogate for symptomatic malaria, and in one study conducted in Mozambique, report of fever in the previous 30 days was associated with confirmation of malaria parasitaemia detection by RDT [20]. However, it must be acknowledged that while report of fever approximates symptomatic disease, it underestimates parasitaemia. Report of fever as a surrogate for symptomatic malaria lacks both the sensitivity and specificity to be relied on as a clinical test, but it may be useful for epidemiologic purposes.

Another potential limitation is that there may have been other important but unmeasured covariates. Under ideal circumstances the following household level variables would have been collected and included in the logistic regression model: precipitation, elevation, temperature, indoor residual spraying, type of door, building material, construction on home, proximity to agriculture/farms, proximity to water, population density, land type, and mosquito species in area. However, the large sample size did allow for the inclusion of many important variables into the regression model without overfitting it, and additional environmental factors were accounted for by performing an ecological comparison of observed prevalence of symptomatic malaria against predicted mosquito suitability.

## Conclusions

Self-reported symptomatic malaria is highly prevalent among children in Zambézia Province, Mozambique, and our findings highlight the need for intensified focus on the poorest and least educated caregivers, who may have the most difficulty seeking malaria diagnosis, care, and treatment for their children.

## Abbreviations

CI: confidence interval
EA: enumeration area
GIS: geographic information system
IRS: indoor residual spraying
ITN: insecticide-treated bed net
MZN: meticais
MOH: Ministry of Health
OR: odds ratio
PMI: President’s Malaria Initiative
RDT: rapid diagnostic test
SCIP: Strengthening Communities through Integrated Programming
USAID: United States Agency for International Development
WAZ: weight-for-age Z-score
WHO: World Health Organization
